# Ecotypes or phenotypic plasticity—The aquatic and terrestrial forms of *Helosciadium repens* (Apiaceae)

**DOI:** 10.1002/ece3.5833

**Published:** 2019-11-25

**Authors:** Tobias Herden, Nikolai Friesen

**Affiliations:** ^1^ Botanical Garden of the Osnabrueck University Osnabrueck Germany; ^2^ Department of Pharmaceutical and Natural Sciences Ministry of Health of the Russian Federation I. M. Sechenov First Moscow State Medical University Moscow Russia

**Keywords:** creeping marshwort, ecotypes, fingerprinting, genetic differentiation, *Helosciadium repens*, phenotypic plasticity

## Abstract

- Morphological and ecological differences of two forms of *Helosciadium repens* are known and described in the literature: aquatic and terrestrial. However, their taxonomic status is currently unknown. The question whether they are genotypically adapted to specific environmental conditions or are those differences a result of phenotypic plasticity is addressed in this study.
- SSR and ISSR data were used to uncover genotypic differences. Data from drought stress experiments (system water content and relative water content of leaves) were used to evaluate the response to water as an environmental factor. The stomatal index of both forms grown under different water treatments was analyzed.
- The principal component analysis of the ISSR data revealed no clustering that would correspond with ecotypes. The diversity parameters of the SSR data showed no significant differences. The aquatic populations showed a tendency toward heterozygosity, while the terrestrial ones showed a bias toward homozygosity. Both forms responded similarly to the changes in water availability, with newly produced leaves after drought stress that were better adapted to repeated drought stress. Stomatal indices were higher in plants from aquatic habitats, but these differences disappeared when the plants were grown in soil.
- The observed responses indicate that the differences between forms are due to phenotypic plasticity.

## Graphical abstract

There are two morphologically distinct forms of *Helosciadium repens* described in the literature. Based on the molecular data (SSR and ISSR fingerprinting) and adaptation experiments on drought stress, we conclude that phenotypic plasticity is causing the differences. *Helosciadium repens* is well adapted to water fluctuations.

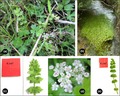

## 1 INTRODUCTION

Most organisms exhibit different phenotypes in response to different environmental factors (Xue & Leibler, 2018). Heterophylly in *Neobeckia aquatica* (Eaton) Greene in response to the temperature and submergence (Amano et al., 2015) and *Arabidopsis thaliana* as a response to light (Mishra et al., 2012) are only two of many examples that can be found in the plant kingdom. Even metabolic changes in the form of carbon fixation can occur such as in the case of *Mesembryanthemum crystallinum* L. (Tallman et al., 1997). These responses are all considered to be evolutionary strategies for adapting to variable environments (Xue & Leibler, 2018). In some extreme cases such as in *Pinus sylvestris* L., the high spectrum of phenotype variability led to the assignment of various names currently recognized as synonyms (The Plant List, 2013). Contrarily to the characteristics, which justify taxa levels, those apparent morphological differences are nonpermanent and disappear when the plants grow under the same conditions.

**Figure 1:**
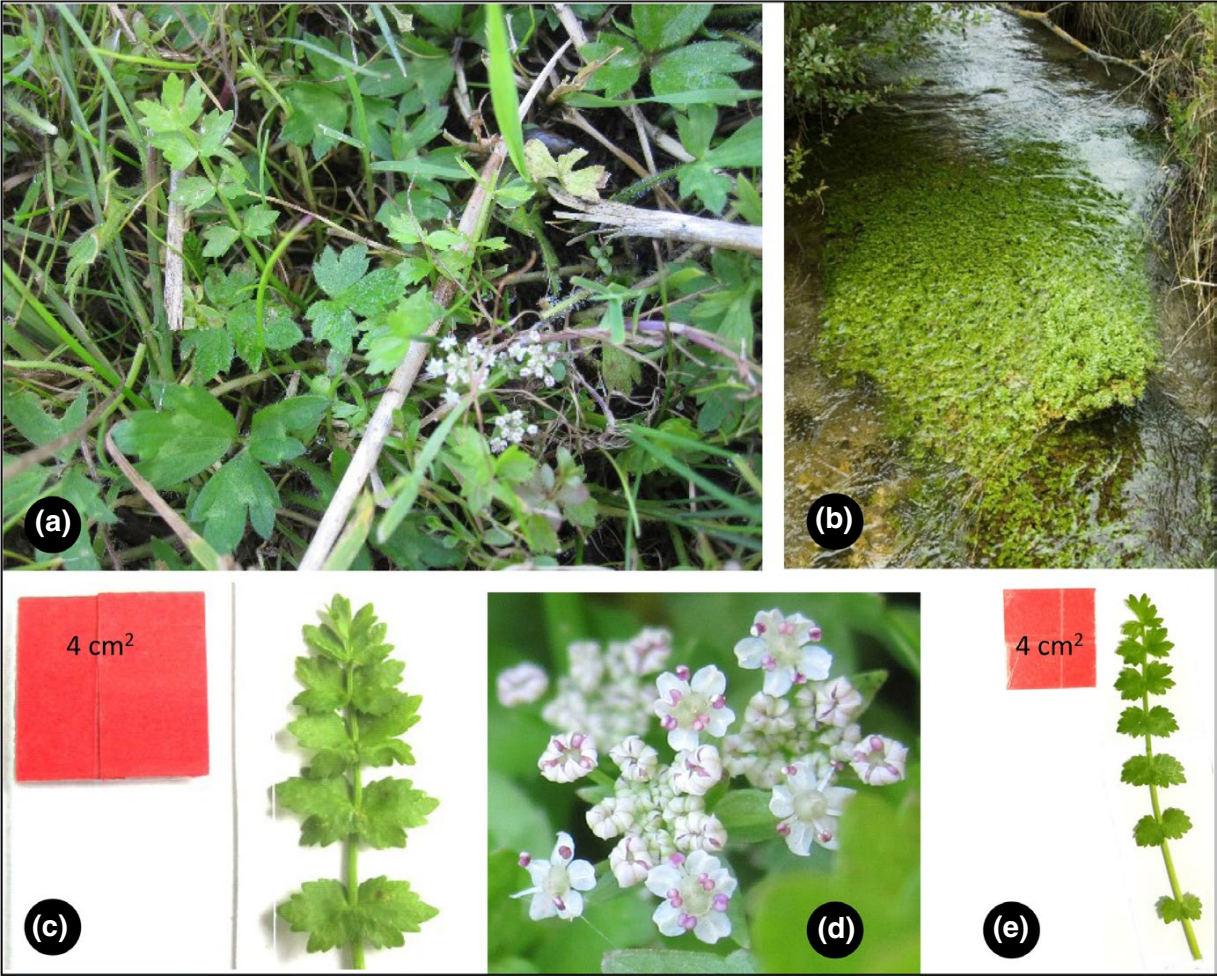
Terrestrial and aquatic forms of *Helosciadium repens* (a) terrestrial form at the natural site, (b) aquatic form at the natural site, (c) leaf of terrestrial form, (d) inflorescence of the terrestrial form, and (e) leaf of the aquatic form

*Helosciadium repens* (Jacq.) W.D.J. Koch (Apiaceae) (Figure 1) is a small perennial herb, growing on alternating wet pastures, littoral zones of trenches and springs (Weber, 1995), and along or in slow running streams (pers. observation). Two forms are known and described from the literature (Casper & Krausch, 1981; Hacker, Voigtländer, & Russow, 2003; NLWKN, 2011; Voightländer & Mohr, 2008). The terrestrial (hereafter *Terr*) form is hemicryptophytic and grows leaves with a length between 10 and 30 cm (Oberdorfer, 1983; Schubert & Vent, 1994). Their stolons can grow to a length between 20 and 30 cm and can as such colonize open patches very quickly (Hacker et al., 2003). The flowers are arranged in an umbel and produce nectar and a schizocarp fruit which releases two seeds per flower (East, 1940; Frank & Klotz, 1990; T. Herden, M. Bönisch, & N. Friesen, unpublished data; Klotz, Kühn, & Durka, 2002; NLWKN, 2011). *Helosciadium repens* also build up soil seed banks (Burmeier & Jensen, 2008). According to the database *BIOLFLOR* (Frank & Klotz, 1990), *H. repens* can self‐fertilize. They, however, cite East (1940) as a reference for this statement. Upon further investigation, we uncover that East (1940) stated that little is known about the self‐fertilization in this taxon (applying to Umbelliferae) and did not mention *H. repens* at all.

The hydrophytic populations or aquatic form (hereafter *Aqu*) can be occasionally found in Southern Germany, Bavaria (pers. observation). The aquatic form tends to exhibit vegetative growth only and does not produce flowers (Casper & Krausch, 1981; Schossau 2000 cited in Hacker et al., 2003; NLWKN, 2011). They can grow leaves up to 40 cm in length (Casper & Krausch, 1981), can colonize waterbodies up to a depth of 60 cm, and their stolons can grow up to a length of 150 cm (Voightländer & Mohr, 2008). They stay immotile due to their roots anchored on driftwood, tree roots, or other aquatic vegetation. The plants do not root in the substrate (pers. observation).

There is scarce information on the two different manifestations in the literature. However, when mentioned, authors address both appearances as different forms of the species, and do not specify what the word “*forms*” means in the corresponding context (Casper & Krausch, 1981; Hacker et al., 2003; NLWKN, 2011; Voightländer & Mohr, 2008). Whether they are genotypically adapted to specific environmental conditions or a result of phenotypic plasticity is thus still unknown. T. Herden, M. Bönisch, & N. Friesen (unpublished data) analyzed 27 populations of *H. repens* in Germany with SSRs and found only low levels of variation within the analysed markers. There we found no genetically based separation into a *Terr* or *Aqu* cluster, suggesting differences due to phenotypic plasticity. However, our sample set was not aimed to address the taxonomic status of both forms. The ecotype hypothesis cannot be excluded based only on these results. Markers may fail to detect quantitative variation for adaptively important traits (Bekessy, Ennos, Burgman, Newton, & Ades, 2003; McKay & Latta, 2002).

If both forms appear to be ecotypes, it can have consequences on the conservation management. Ex situ conservation management for aquatic forms needs to be adapted as well as conservation at the natural sites. Additionally, this information might be interesting for plant breeders as both ecotypes may harbor specific traits of interest.

This comparison study aimed to answer the question of the taxonomical status of both forms by using simple sequence repeats (SSR) and intersimple sequence repeats (ISSR) data on a balanced sample set.

Additionally, the adaptation of both forms to drought stress was studied by measuring the relative water content (RWC) of leaves, system water content, and water loss during drought stress conditions. The stomatal index (SI) was measured for different water treatment levels. A small scale experiment was set up, to determine whether *H. repens* is capable of self‐fertilization.

## 2 MATERIAL AND METHODS

### 2.1 Genetic analysis

#### 2.1.1 SSR analysis

SSR or microsatellites are short stretches of repeated short nucleotide motifs. These motifs typically consist of mono‐, di‐, and tri‐nucleotides, but even longer ones can be found. The repetitions of the motifs are mainly <100 base pairs (bp) long and can be found in all genomes (Tautz, 1989). They can show side‐specific length variation because of the occurrence of different numbers of repeat units (Morgante & Olivieri, 1993). Most of these length differences are caused by the slippage effect during replication and accumulate over time (Tautz & Schlötterer, 1994). Using the polymerase chain reaction (PCR), with specific primer pairs flanking a specific microsatellite, it is possible to amplify and measure the exact bp length of a microsatellite. SSR markers are considered to be a reliable system for diversity studies as they are codominant and multiallelic (Baldwin, Pither‐Joyce, Wright, Chen, & McCallum, 2012; Fu, Kong, Yingxiong, & Cameron, 2005; Geethanjali, Anitha Rukmani, & Rajakumar, 2018; Park, Lee, & Kim, 2009; Yasodha et al., 2018). They are neutral markers and are thus usually not subjected to natural selection (Holderegger, Kamm, & Gugerli, 2006; Kimura, 1983).

The data from T. Herden, M. Bönisch, & N. Friesen (unpublished data) were evaluated to investigate genetic differences between *Aqu* (16R, 19R, 20R, 21R, 22R, 24R, and 25R) and *Terr* (1R, 5R, 8R, 9R, 10R, 12R, 18R, and 27R) populations (Table 1). Counts for allelic richness, fixation index (*F*‐Index), inbreeding coefficient *F*_is_, private alleles, rare alleles, single locus genotypes (SLG), multilocus genotypes (MLG), and numbers of alleles were taken from the data analysis of T. Herden, M. Bönisch, & N. Friesen (unpublished data) (Table S2).

**Table 1:** Provenances of the analysis populations (modified after T. Herden, M. Bönisch, & N. Friesen (unpublished data))

Lab‐ID	GE‐Sell ID	State	District	Commune	Form
1R	MV‐GC‐20120912‐1400	MV	Demmin	Meesiger	Terr
5R	MV‐DS‐20131029‐1030	MV	Müritz	Alt Schwerin	Terr
8R	NRW‐DB‐20150818‐1831	NW	Paderborn	Delbrück	Terr
9R	NI‐OM‐20150812‐0955	NI	Diepholz	Hüde	Terr
10R	SH‐TIV‐20150902‐0900/0910/0920	SH	Plön	Blekendorf	Terr
12R	Bbg‐SE‐20150723‐1634	BB	Havelland	Seeblick	Terr
16R	BY‐GAP_FARC‐20151021‐1004	BY	Garmisch‐Partenkirchen	Farchant	Aqu
18R	BY‐KEH_NIED‐20150908‐1005	BY	Kelheim	Langquaid	Terr
19R	BY‐KF_KAUF‐20150814‐1012	BY	Kaufbeuren	Kaufbeuren	Aqu
20R	BY‐LL_BISC‐20160828‐1022	BY	Landsberg am Lech	Dießen am Ammersee	Aqu
21R	BY‐MB_TRAC‐20150811‐1002	BY	Miesbach	Fischbachau	Aqu
22R	BY‐MB_TRIN_20150802‐1003	BY	Miesbach	Kreuth	Aqu
24R	BY‐MN_SALG‐20150804‐1019	BY	Unterallgäu	Salgen	Aqu
25R	BY‐MUE_MARS‐20150829‐1027	BY	Mühldorf a. Inn	Maitenbeth	Aqu
27R	BY‐TS_WINK‐20151114‐1001	BY	Traunstein	Übersee	Terr

Notes

GE‐Sell ID = reference IDs used in the project GE‐Sell, states = federal states of Germany (BB = Brandenburg, BY = Bavaria, MV = Mecklenburg‐West Pomerania, NI = Lower Saxony, NW = North Rhine‐Westphalia, SH = Schleswig Holstein, ST = Sachsen Anhalt), form = form of *H. repens* (Terr—terrestrial; Aqu—aquatic).

#### 2.1.2 ISSR analysis

Intersimple sequence repeats (ISSR) are regions between microsatellite loci. In a PCR, only one primer containing an SSR motif is used, which amplifies multiple fragments with various length (Reddy, Sarla, & Siddiq, 2002; Zietkiewicz, Rafalski, & Labuda, 1994). Only regions between adjacent, inversely oriented SSRs are thus amplified (Zietkiewicz et al., 1994). Usually, the PCR products are visualized on an agarose gel, and the banding pattern is transformed into a binary matrix. Every band is treated as a single trait. By analyzing the matrix, kinship relations can be computed. Polymorphism can be detected due to mismatches in the priming site (changes in the SSR where the primer binds) or differences in length of the amplified sequences (Zietkiewicz et al., 1994). This method has been widely used for decades in population genetic studies and studies to characterize genetic divergence among species (Andiego et al., 2019; Kumar, Mishra, Singh, & Sundaresan, 2014; Reddy et al., 2002; Schlotteröer, Amos, & Tautz, 1991; Zietkiewicz et al., 1994).

DNA isolates were taken from T. Herden, M. Bönisch, & N. Friesen (unpublished data). An agarose gel documentation with 47 lanes was used. Three individuals from each population (eight *Terr*—1R, 5R, 8R, 9R, 10R, 12R, 18R, and 27R and seven *Aqu* populations—16R, 19R, 20R, 21R, 22R, 24R, and 25R) were chosen for further investigation (Table 1). The isolated DNA was used directly in a PCR with 10 µl Biozym red HS Taq master mix (Biozym Scientific GmbH), 1 µl of corresponding primer (Table S1), and 1 µl DNA template in a final volume of 20 µl. PCR products were checked on an agarose gel. The bands were scored independently as either present (1) or absent (0) and summarized in a matrix. Polymorphism information content (PIC) values were calculated using the formula described previously in Roldan‐Ruiz, Dendauw, Bockstaele, & Depicker, 2000. A principal component analysis (PCA) was performed using the function *dudi.pca* from the R package *ade4* (Bougeard & Dray, 2018; Chessel, Dufour, & Thioulouse, 2004; Dray & Dufour, 2007; Dray, Dufour, & Chessel, 2007).

### 2.2 Self‐fertilization test

Plants from two populations that were currently available (nine individuals from 9R and nine from a population from Austria) were potted in trays. These were then isolated from potential pollinators using transparent plastic hoods with *Drosophila* impermeable mesh for airflow. One control from each population was potted outside of the isolation hoods. The isolated individuals were pollinated by hand with their pollen. At the end of their vegetation period, the seeds were collected. Seeds were drawn randomly for germination tests.

### 2.3 Dry stress experiment

Stolons from 15 *Terr* plants (population 9R) were potted in 10 × 10 cm pots (the stolon was approximately 5 cm long with two leaves). For substrate, 173 g of “Einheitserde Special” (Einheitserdewerke Werkverband e.V., Sinntal‐Altengronau, Germany) was used. Plants were grown for three weeks in a greenhouse to ensure that they have rooted successfully. During that time, all pots stood in trays filled with water to ensure that they were watered to their maximum water capacity. They were treated with extra light using one unit of the *KIND LED L600 grow light* (Santa Rosa, CA), until the start of the experiment. All plants were weighted (system water content (SWC) = weight of soil, pot, and plant) just before they were put into a climatic chamber (maximum run time for every run: 20 days, day temperature: 33°C; night temperature: 22°C; light: 14 hr; dark: 10 hr; rel. humidity >80%). The pots were weighed daily during the runs. During the experiment, the pots were not watered. To ensure that all plants grew under the same condition, the pots' positions in the chamber rotated every day. If plants lost all their leaves due to wilting, they were taken out of the chamber and watered immediately to their maximum water capacity, to prevent the loss of study material.

All plants recovered during a recuperation period of three weeks in the same greenhouse conditions as mentioned above. The experiment was then repeated with the same plants to assess potential adaptation.

The same experiment was conducted with plants from *Aqu* populations (population 16R, 22R, 24R), which were grown in soil for a time period of one year. For that, five individuals were collected at three natural sites (with the maximum distance between each sample) and cuttings were used in the experiment. For every plant, the results were statistically evaluated by one‐way analysis of variance (ANOVA), using the software R (R Core Team, 2017).

At the beginning of each run, one leaf from every plant was used to measure the RWC. For that, the weight (W) of a freshly harvested leaf was measured and put in a 50‐ml centrifuge tube with 5 ml of distilled water for rehydration. As Arndt, Irawan, and Sanders (2015) already indicated, rehydration by floating leads to erroneous RWC estimates. Therefore, the leaves were put petiole first in the distilled water, making sure that the water level did not reach the lowest pair of leaflets. They were rehydrated for three hours in darkness under room temperature conditions, and the turgid weight (TW) was measured afterward. All leaves were left in a dry chamber with 10% relative air humidity overnight and weighted afterward to measure the dry weight (DW). The RWC was calculated using the formula of Weatherley (1950). The measurement was also carried out with leaves that exhibit a complete loss of turgor pressure. The system water loss (SWL) was calculated (SWL = (1 − SWC_end_/SWC_start_) × 100).

Tests for significance were made with the geom_signif function using the R package *ggplot2*, and plots were drawn using the function *ggplot* from the R package *ggplot2* (Wickham & Chang, 2018).

### 2.4 Stomatal index

To estimate the SI, nail polish impressions from the epidermis were made (as described in Miller & Ashby, 1968) from plants cultivated ex situ in the Botanical Garden of Osnabrueck, Germany. Ten impressions from the upper surface were made from all leaflet pairs of a leaf, to test whether there are significant differences between each leaflet pair. The same was done for the lower leaf surface. Pictures of impressions were made using a transmitted light microscope under 400× magnification. Stomata counts (SC) and epidermis cell counts (EC) were quantified (guard cells were treated as a part of the stomatal apparatus). The observed surface area was measured (A), and the stomatal density (SCD), as well as the epidermal cell density (ECD), was calculated. Three pictures were taken from every leaflet pair, and the quantifications of the SC and EC were averaged. The SI was calculated for every leaflet pair using the equation from Salisbury (1928).

The SI was calculated for two different water treatment levels for every form: *Terr*—terrestrial form growing in pots with drainage with local weather conditions, *T‐Wet*—terrestrial form watered to their maximum water capacity, *Aqu*—aquatic form growing under aquatic conditions, and *A‐T*—aquatic form potted in soil and growing under the same conditions as *T‐Wet*.

## 3 RESULTS

### 3.1 Genetic analysis

#### 3.1.1 SSR analysis

**Figure 2:**
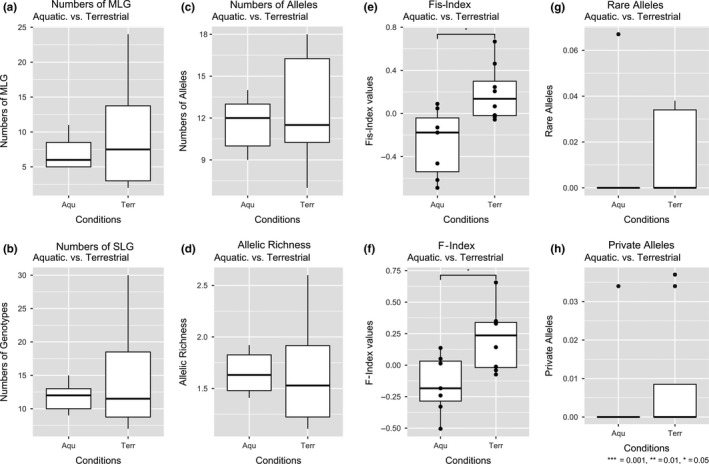
Comparison between aquatic and terrestrial populations of *Helosciadium repens* in Germany using diversity parameters from the SSR analysis. (a) Numbers of multilocus genotypes, (b) numbers of genotypes, (c) numbers of alleles, (d) allelic richness, (e) inbreeding coefficient *F*_is_‐Index, (f) fixation index *F*, (g) counts of rare alleles, and (h) counts of private alleles. Asterisks are indicating significance levels

There were no significant differences in the numbers of MLG, SLG, alleles, allelic richness, rare alleles, and private alleles between *Terr* and *Aqu* plants (Figure 2a–d,g,h). As T. Herden, M. Bönisch, & N. Friesen (unpublished data) showed, there is no genetically based separation into a *Terr* or *Aqu* cluster. However, there were significant differences (*p* < .01) in the *F*‐ and *F*_is_‐Indices between both forms. The *F*‐Index values for the *Aqu* populations were mostly negative indicating an excess of heterozygosity. There were only three populations (20R, 22R, and 16R) with positive values. Most of the *Terr* populations had positive *F*‐Index values indicating an excess of homozygosity. Only five populations (2R, 10R, 11R, 12R, and 18R) showed negative values (Figure 2e). The same was true for *F*_is_‐Index values (Figure 2f). Only two *Aqu* populations had positive values (20R and 22R) and one exhibited a *F*_is_‐Index value of zero (23R). Five *Terr* populations (3R, 10R, 11R, 12R, and 18R) had negative values (Table S2). The rest of the *Terr* populations exhibited positive *F*_is_‐Index values.

#### 3.1.2 ISSR analysis

Only eight out of 26 tested ISSR markers produced evaluable polymorphic bands. A total of 108 bands were amplified out of which 64 were polymorphic, and 42 were monomorphic bands (Table S1). The percentage of polymorphic bands (*P*%) per primer ranged from 77.8% in UBC813 to 38.9% in UBC834. The average percentage of the polymorphic band was 60.7%. PIC values spanned from 0.4409 in UNC810 to 0.2647 in HB15 (Table S1).

**Figure 3:**
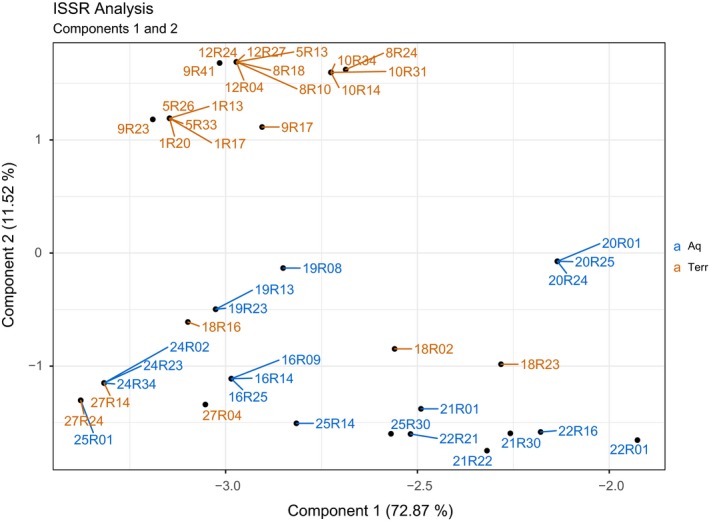
Principal component analysis of the ISSR data of eight terrestrial and seven aquatic populations. Blue = Aq=aquatic population, orange = Terr=terrestrial populations; Lab IDs = first digits including the letter R (see Table 1); individuals = digits after the letter R

The first three components of the PCA explained 87.87% of the data (comp. 1:72.87%, comp. 2:11.52%, and comp. 3:2.67%) (Figure 3). Two distinct clusters were visible. One was composed of Bavarian populations and one of the populations from northern Germany. This partitioning coincides with the SSR analysis of T. Herden, M. Bönisch, & N. Friesen (unpublished data). Separation into *Terr* or *Aqu* clusters was not observed.

Both analyses (SSR and ISSR) showed congruent results, namely a split between Northern and Southern populations (Figure 3) (T. Herden, M. Bönisch, & N. Friesen, unpublished data).

### 3.2 Self‐fertilization test

There was an evident difference in the number of seeds between the isolated and their control pots. However, due to high humidity in the isolated trays, some of the inflorescences and infructescences started to rot. Therefore, a test for statistical significance was not possible. Nevertheless, the isolated plants produced seeds when fertilized with their pollen. Randomly selected seeds were able to germinate.

### 3.3 Dry stress experiment

#### 3.3.1 Terrestrial plants

**Figure 4:**
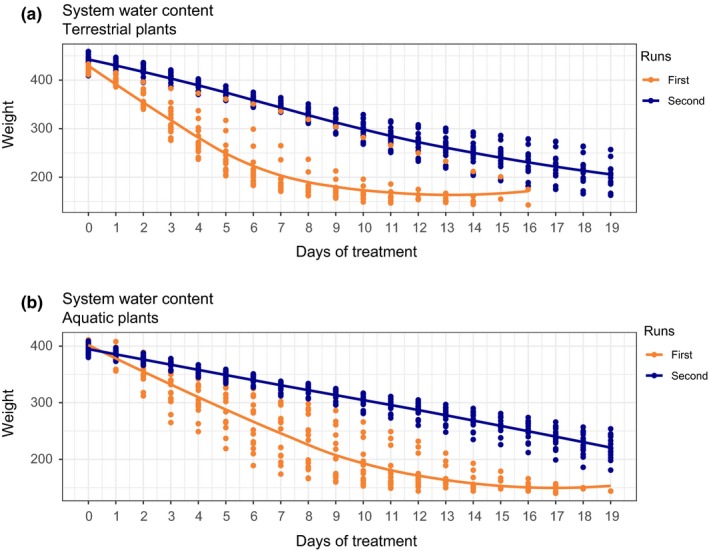
Daily system water content of the first and second runs during the drought stress experiment. (a) Terrestrial plants, (b) potted aquatic plants; orange lines represent smoothed conditional means of the first runs, and blue lines represent smoothed conditional means of the second run

During the first run of the *Terr* plants, none of the plants endured the scheduled time of 20 days without a complete loss of leaves. About 46% of the plants dropped all leaves during the first nine to 11 days. Two plants shed all its leaves after 16 days (Figure 4a). On average, there was a SWL of 64% at which plants lost all their leaves. After 16 days, the control pot had an SWL of 51%. The relationship of the variables was explained best with a polynomial regression (0.9902 < adjusted *R* ^2^ < 0.9993, median: 0.998) instead of a linear regression (0.8663 < adjusted *R* ^2^ < 0.9917, median: 0.9167) (adjusted *R* ^2^, slopes (*b*), intercept (*a*), and residual standard deviation (res. *SD*) are given in Figure S1a,b). Only plant VI had an even water loss which was comparable to the linear regression (Figure S1a).

The new leaves that grew back during the recovery period were smaller and stiffer.

In the second run, all plants endured the scheduled time of 20 days without a complete loss of leaves (Figure 4a). The plants showed signs of withering, after an average SWL of 55% (39%–65%). The control pot had an SWL of 38%. Overall, the SWC was significantly higher during the second run. The adjusted *R* ^2^s were higher than 0.98, except for plants II, IV, and V (>0.97) (Figure S1a). The relationship of the variables during the second run almost fits a linear regression in all investigated plants (Figure S1a,b). The slopes of the linear regressions were between −16.6 and −8.1 (median: −12.76).

Figure 4a shows the smoothed conditional means of all plants during the first and second runs. The curve is, except for the slope (*b*_control_ = −7.9948, *b*_median_ _=_ −12.7554), comparable to the one from the control pot (Figure S1b plant control).

**Figure 5:**
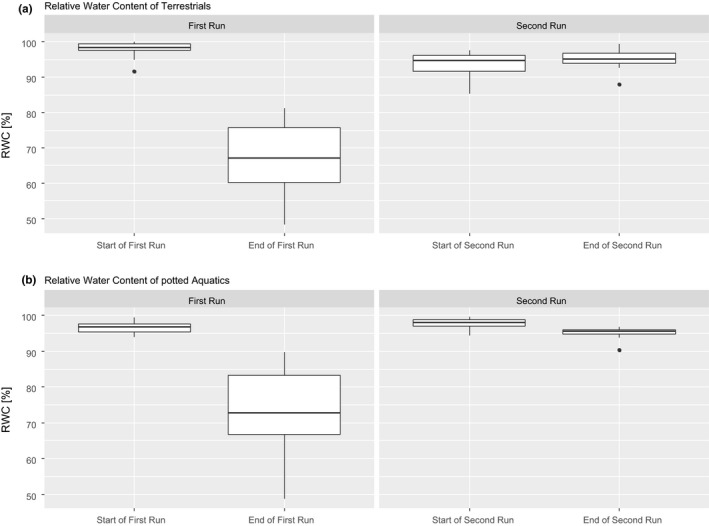
Relative water content at the start and the end of the first and second runs during the drought stress experiment. (a) Terrestrials, (b) potted aquatics

The RWCs of leaves at the start of run one (with full turgor pressure) and the end of the run one (complete loss of turgor pressure) were significantly different (Figure 5a). The leaves lost on average 31.44% (lowest: 15.1%; highest: 50.49%) of water. In run two, the RWCs did not differ significantly between the beginning and the end of the run (Figure 5a). The leaves had a negative water loss and gained on average 1.76% (lowest: −13.75%; highest: 3.75%) of water.

#### 3.3.2 Potted aquatics plants

During the first run, only one plant out of 15 endured the scheduled time of 20 days without a complete loss of leaves (Figure 4b). At day 15, 53% of the plants lost all their leaves. The average SWL was 63% and ranged from 61% to 65%. The control pot had an SWL of 46%.

The adjusted *R* ^2^ for the linear regression for the SWC curves of each plant was between 0.9929 and 0.8864 (median: 0.9549) with a res. *SD* between 28.49 and 7.279 (median: 17.61). The curves of plant III, VIII, and X are very close to that of the linear regression with the adjusted *R* ^2^ > 0.98 and the res. *SD* < 9.2 (Figure S1c,d). However, the relationship of the variables was best explained with polynomial regression (adj. *R* ^2^: 0.9893–0.9967, median: 0.9945; res. *SD*: 9.092–4.957, median: 6.479) (Figure S1c,d).

The new leaves that grew back during the recovery period were smaller and stiffer.

In the second run, all plants endured the scheduled time of 20 days without a complete loss of leaves (Figure 4b). The plants showed signs of withering at an average SWL of 45% (36%–57%). The control pot had an SWL of 35%. The adj. *R* ^2^ was between 0.9968 and 0.9991 with a res. *SD* between 3.464 and 1.528. The curves fit the linear regression (Figure S1c,d). For plants III and VIII, the curves fit the linear regression best in the second run (Figure S1c). The slopes of the linear regressions were between −11.92 and −7.13 (median: −9.08). The slope of the linear regression of the control pot was −6.62 (Figure S1d plant control).

Figure 4b shows the smoothed conditional means of all plants during the first and second runs. The curve is, except for the slope (*b*_control_ = −6.62, *b*_median_ = −9.08), comparable to the one from the control (Figure S1d plant control).

The RWCs between leaves at the start of the run one (with full turgor pressure) and those at the end of the run one (complete loss of turgor pressure) differed significantly (Figure 5b). The leaves lost on average 23.95% (lowest: 7.33%; highest: 49%) of water. In run two, the RWCs were again significantly different when comparing the beginning and the end of the run. The leaves lost on average 2.51% (lowest: −0.3%; highest: 6.1%) of water. The difference in water loss between both runs was significant (*p* < .001, data not shown).

The RWC at the start of both runs was significantly different, comparing both conditions (*Aqu* and *Terr*). On average, the differences were 1.28% in the first run and 4.3% in the second run. At the end of both runs, the RWCs in both conditions were not significantly different anymore (*p* < .001, data not shown).

### 3.4 Stomatal index

**Figure 6:**
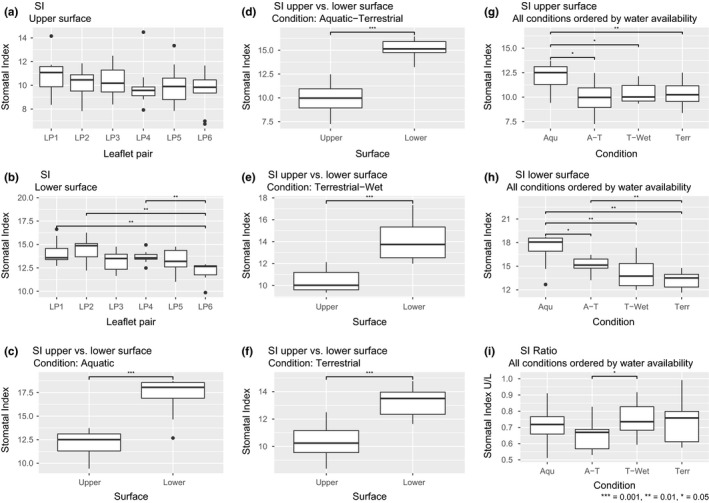
Comparison of the stomatal index (SI). (a) SI from the upper surfaces of different leaflet pairs, (b) SI from the lower surfaces of different leaflet pairs, (c) SI comparison of the upper and lower surface of aquatic plants, (d) SI comparison of the upper and lower surface of potted aquatic plants, (e) SI comparison of the upper and lower surface of terrestrial plants grown in wet conditions, (f) SI comparison of the upper and lower surface of aquatic plants, (g) SI comparison of the upper surface of all conditions, (h) SI comparison of the lower surface of all conditions, (i) comparison of the SI ratio between the upper and lower surfaces of all conditions. Asterisks are indicating significance levels

There were no significant differences between the different leaflet pairs in a leaf (Figure 6a,b). In all conditions (*Aqu*, *A‐T*, *T‐Wet*, and *Terr*), the SI of the upper surface was significantly lower than the SI from the lower surface (*p* < .001) (Figure 6c–f). On the upper surfaces, the SI was significantly higher for *Aqu* than all other conditions with different levels of significance (Figure 6g). There were no significant differences between conditions *A‐T*, *T‐Wet*, and *Terr*.

On the lower surfaces, the SI of *Aqu* was significantly higher (with different levels of significance) in comparison with the SI of plants grown under other conditions (Figure 6h). There was a significant difference between the SI of *AT* and *Terr*. However, there was no significant difference between both forms grown under the same condition (*A‐T* and *T‐Wet*).

The ratio (SI upper/SI lower) between the upper and lower surfaces for each condition was analyzed (Figure 6i). The only significant difference was detected between *A‐T* and *T‐Wet* (.01 < *p* < .05).

### 3.5 General observations

Five cuttings from every *Aqu* populations were potted and the rest grown in small trays with water. All plants, in the trays with water and the pots, build inflorescences and infructescences.

## 4 DISCUSSION

Two main results derived from this study: (a) The analyses of the SSR and ISSR data showed similar outcomes and no significant separation into ecotypes; (b) the differences in morphological characters of the two forms faded when plants were grown under the same conditions.

### 4.1 Genetic comparison

Both fingerprinting methods (SSR and ISSR) together portray the genetic diversity of the entire genomes of all investigated individuals. Nevertheless, most populations can be genetically told apart from each other; both forms are not genetically differentiated (Figures 2 and 3). Therefore, a taxonomical division based on molecular data is not justified.

The only significant difference recovered from the genetic data was from the *F*‐statistics (Figure 2e,f; Table S2). The heterozygote excess, revealed by a negative *F*_is_, can be caused by asexual propagation (Stoeckel et al., 2006). Four out of the seven *Aqu* populations exhibited negative *F* and *F*_is_ values. These findings confirm the observations that these populations tend to grow clonally (Casper & Krausch, 1981; Schossau 2000 cited in Hacker et al., 2003; NLWKN, 2011). However, three of them have positive *F*‐statistic values. A heterozygote deficiency (homozygote excess) is revealed by positive *F*_is_ values and can be caused by self‐fertilization. This is mainly the case in the *Terr* populations.

In *Aqu* populations, most of the leaves are partially submerged due to floating (pers. observation). When leaves are submerged, they encounter an oxygen shortage (Mommer & Visser, 2005). Hypoxia triggers the ethylene production and thus the adjustments to the submerged conditions such as development of aerenchyma (Drew, Jackson, Giffard, & Campbell, 1981; Gunawardena, Pearce, Jackson, Hawes, & Evans, 2001; Jackson & Armstrong, 1999; Jackson, Fenning, Drew, & Saker, 1985; Kordyum, Kozeko, Ovcharenko, & Brykov, 2017; Yamauchi, Shimamura, Nakazono, & Mochizuki, 2013) or submergence‐acclimated leaf forms (Kuwabara, Ikegami, Koshiba, & Nagata, 2003; Kuwabara, Tsukaya, & Nagata, 2001). In the case of *H. repens*, it possibly inhibits the flowering as it does in *Ipomoea nil* (L.) Roth (Suge, 1972; Wilmowicz, Kęsy, & Kopcewicz, 2008) or in *Xanthium pungens* Wallr. (Abeles, 1967). The plants in the tray were in contact with the bottom and were thus able to sustain upright leaves above the water surface. Due to fluctuations in the water level at the natural sites, the very similar conditions can occur possibly leading to infrequent flowering. Burmeier and Jensen (2008) observed that seeds were able to germinate even under water. Therefore, seed recruitment during low water seems possible and could explain the positive *F*_is_ values.

However, these interpretations remain largely hypothetical and constitute a basis for further research.

### 4.2 Morphological comparison

#### 4.2.1 Drought stress

Both forms undoubtedly adapted between the first and the second runs (Figure 4). This adaptation is also visible in the RWC values of the leaves (Figure 5). During the second run, the RWC values of the leaves did not drop as much as in the first runs (Figure 5). In some cases, the leaves even gained water and plants grew new leaves during the run (pers. observation).

#### 4.2.2 Stomatal index

There were no significant differences between forms (Figure 6). One could interpret the differences in the SI of the upper surface of all conditions as a plastic reduction in SI caused by reduced water availability (Figure 6h). The difference in the ratio of upper and lower surface SI between *A‐T* and *T‐Wet* was likely due to the variation in the data and would probably disappear if more repetitions were carried out (Figure 6i). Had this been a genotypic trait, both extreme conditions (*Aqu* and *Terr*) would have shown differences in the SI.

## 5 CONCLUSION

In general, neither molecular data nor the results from water‐manipulating experiments alone can rule out the hypothesis of ecotypes. Molecular markers may fail to detect differences (Bekessy et al., 2003), and there could be other ecological factors in which the two forms behave differently. Billet, Genitoni, and Bozec (2018) analyzed aquatic and terrestrial morphotypes of *Ludwigia grandiflora* (Michx.) Greuter & Burdet and based on morphological traits they found that the terrestrial morphotype outcompetes the aquatic one. However, they did not perform molecular analyses; thus, the molecular basis of *L. grandiflora* adaptation remains unknown.

Ecotype hypotheses can be addressed only when morphology as well as genetic foundation studies is combined (McKay & Latta, 2002). In a study on *Alternanthera philoxeroides* (Mart.) Griseb., Geng et al. (2007) used molecular data (ISSR) and common garden experiments to test the ecotypes hypotheses for aquatic and terrestrial forms. Their data supported, however, the plasticity hypothesis. For *Coccothrinax argentata* (Jacq.) L.H.Bailey, Davis, Lewis, Francisco‐Ortega, and Zona (2007) found minute differences in the ISSR analysis between the mainland and insular populations. However, they found a great deal of plasticity in the traits included in the study that do not support a separation into different taxa. In *Ageratina adenophora* (Spreng.) R.M.King & H.Rob., the authors found evidence for phenotypic plasticity after checking 16 populations with ISSR and common garden experiments (Zhao, Yang, & Xi, 2012). Noel, Machon, and Porcher (2007) analyzed *Ranunculus nodiflorus* L. populations in France with microsatellites and common garden experiments. They found no genetic diversity and strong evidence favoring phenotypic plasticity.

Since our molecular data provide strong evidence against the ecotype hypothesis and the morphological differences disappeared during a simple drought stress experiment, the results can only lead to one explanation: phenotypic plasticity. Moreover, the drought stress experiment showed that plants that experienced drought stress performed better when subjected to drought stress again. This adaptive plasticity in this species enables it to endure short periods of drought stress and periods of water stress (Longa, 2019). It also gives the plants an advantage over competitors in zones of water fluctuations such as wet pastures and littoral zones, where this species naturally occurs. The ability of self‐fertilization may benefit *H. repens* in environments where pollinators are scarce.

## CONFLICT OF INTEREST

None declared.

## AUTHOR CONTRIBUTIONS

T.H. and N.F. conceived the ideas and designed methodology; N.F. supervised the study; T.H. did the laboratory work, conducted the experiments, and collected and analyzed the data; T.H. led the writing of the manuscript. Both authors contributed critically to the draft. The authors have no conflicts of interest to declare.

### OPEN DATA BADGE

This article has earned an https://openscience.com for making publicly available the digitally‐shareable data necessary to reproduce the reported results. The data is available at https://doi.org/10.5061/dryad.m0cfxpnzg.

## Data Availability

Data is available under https://doi.org/10.5061/dryad.m0cfxpnzg.
