# Supplementary figures and images for: Ecotypes or phenotypic plasticity—The aquatic and terrestrial forms of Helosciadium repens (Apiaceae)
Source: Ecol Evol. 2019 Nov 25;9(24):13954–65. doi: 10.1002/ece3.5833 (PMC6953667; doi:10.1002/ece3.5833)

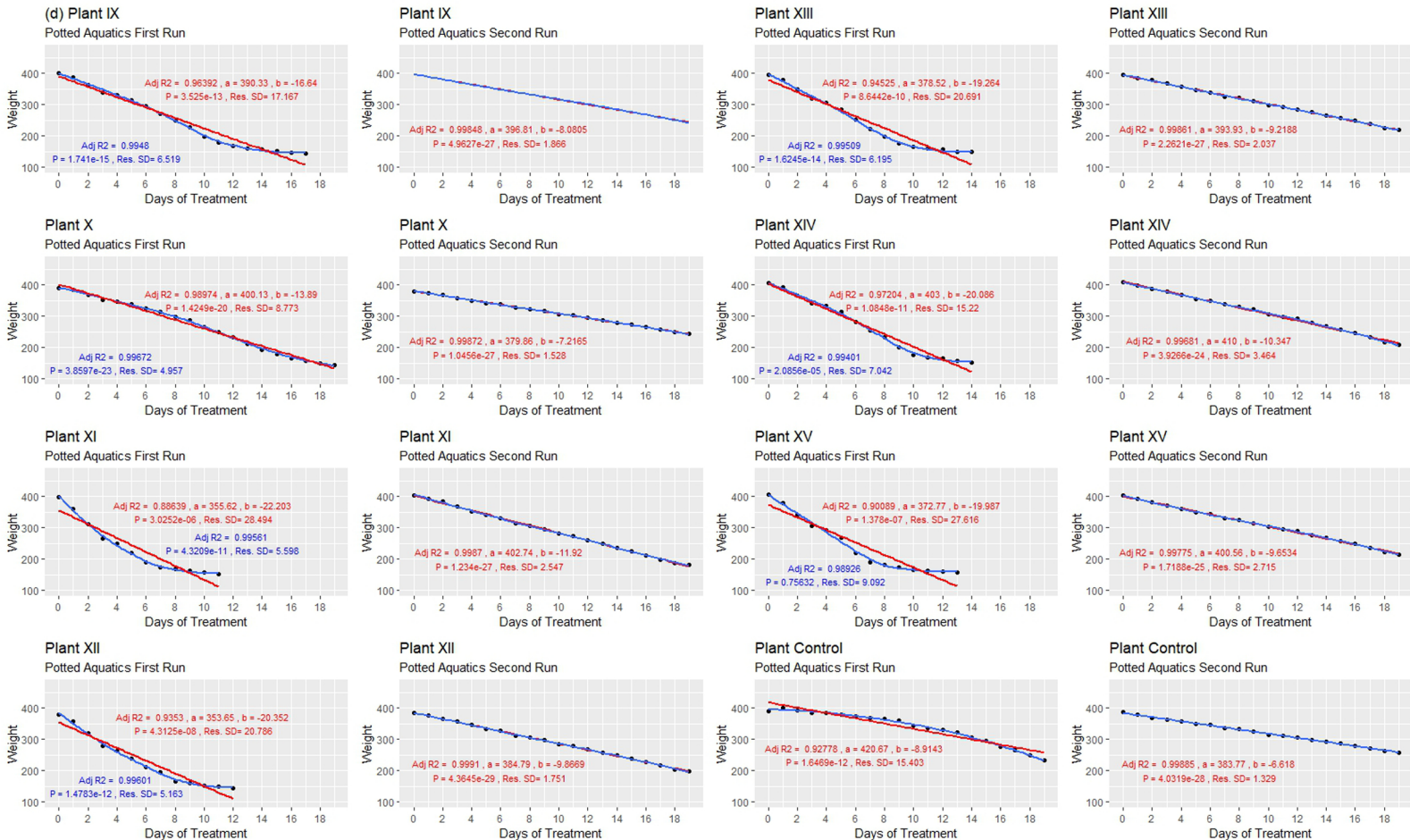

Supplement: Supplementary file 1 [file ECE3-9-13954-s001.zip › ece35833-sup-0004-FigS1d.pdf]

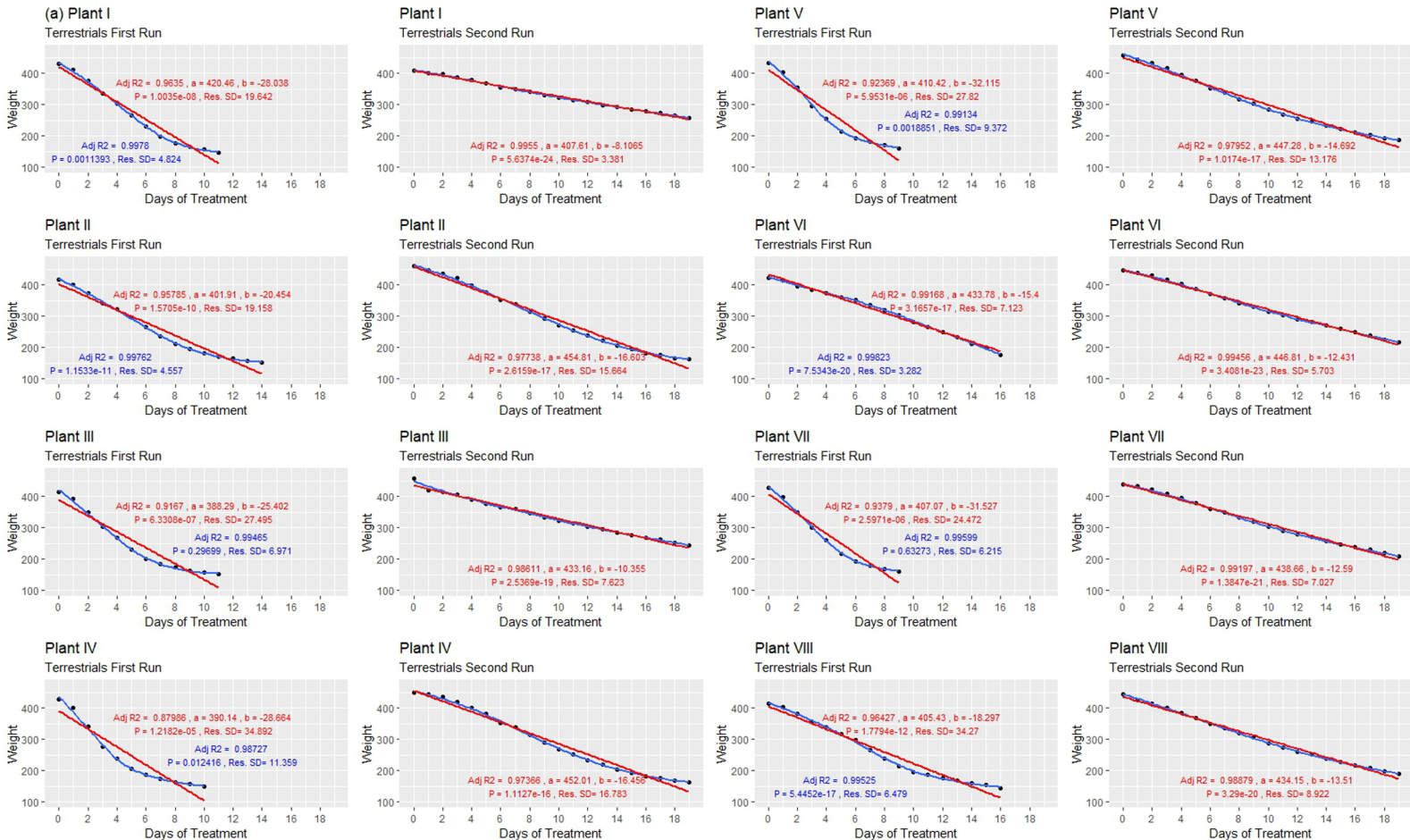

Supplement: Supplementary file 1 [file ECE3-9-13954-s001.zip › ece35833-sup-0001-FigS1a.pdf]

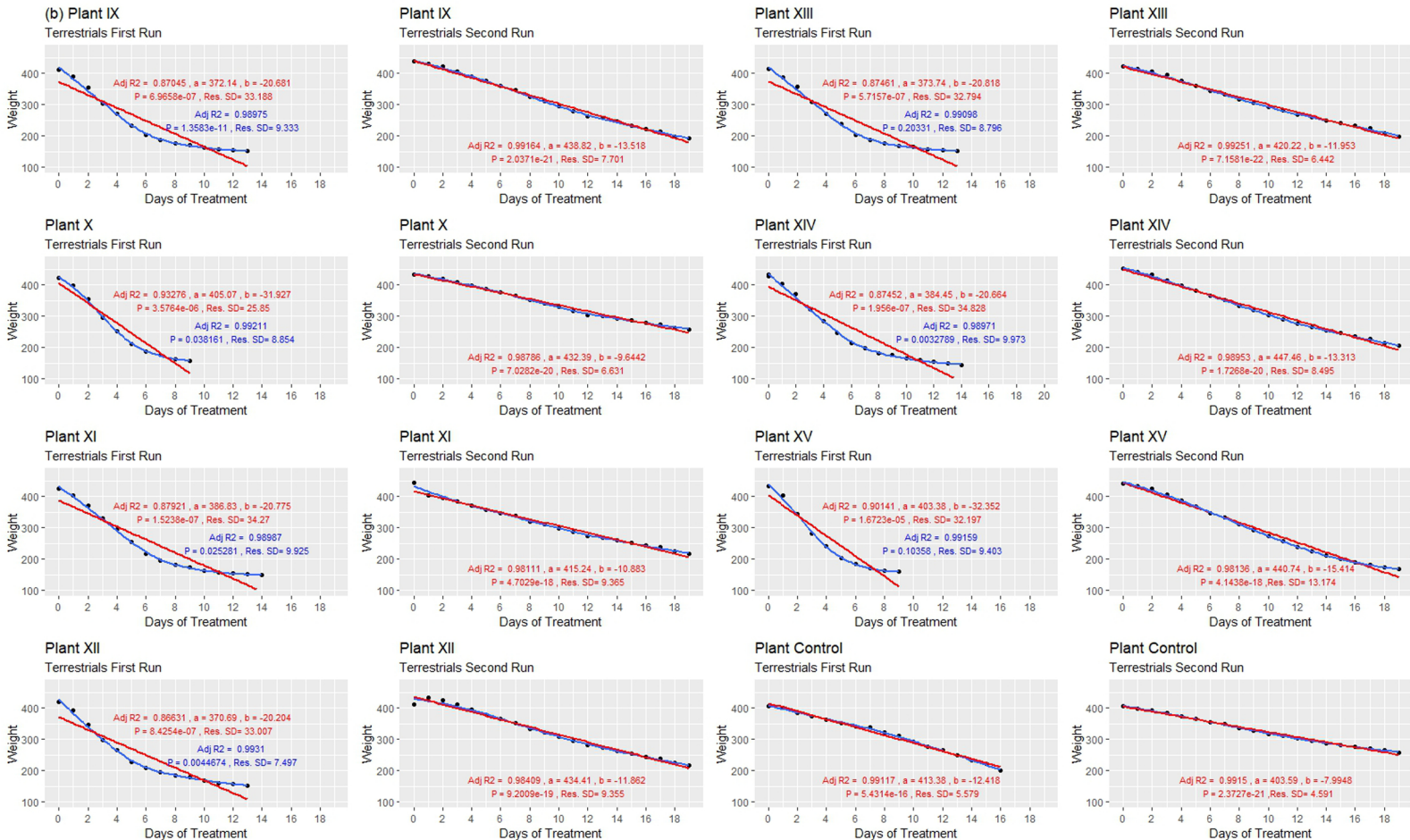

Supplement: Supplementary file 1 [file ECE3-9-13954-s001.zip › ece35833-sup-0002-FigS1b.pdf]

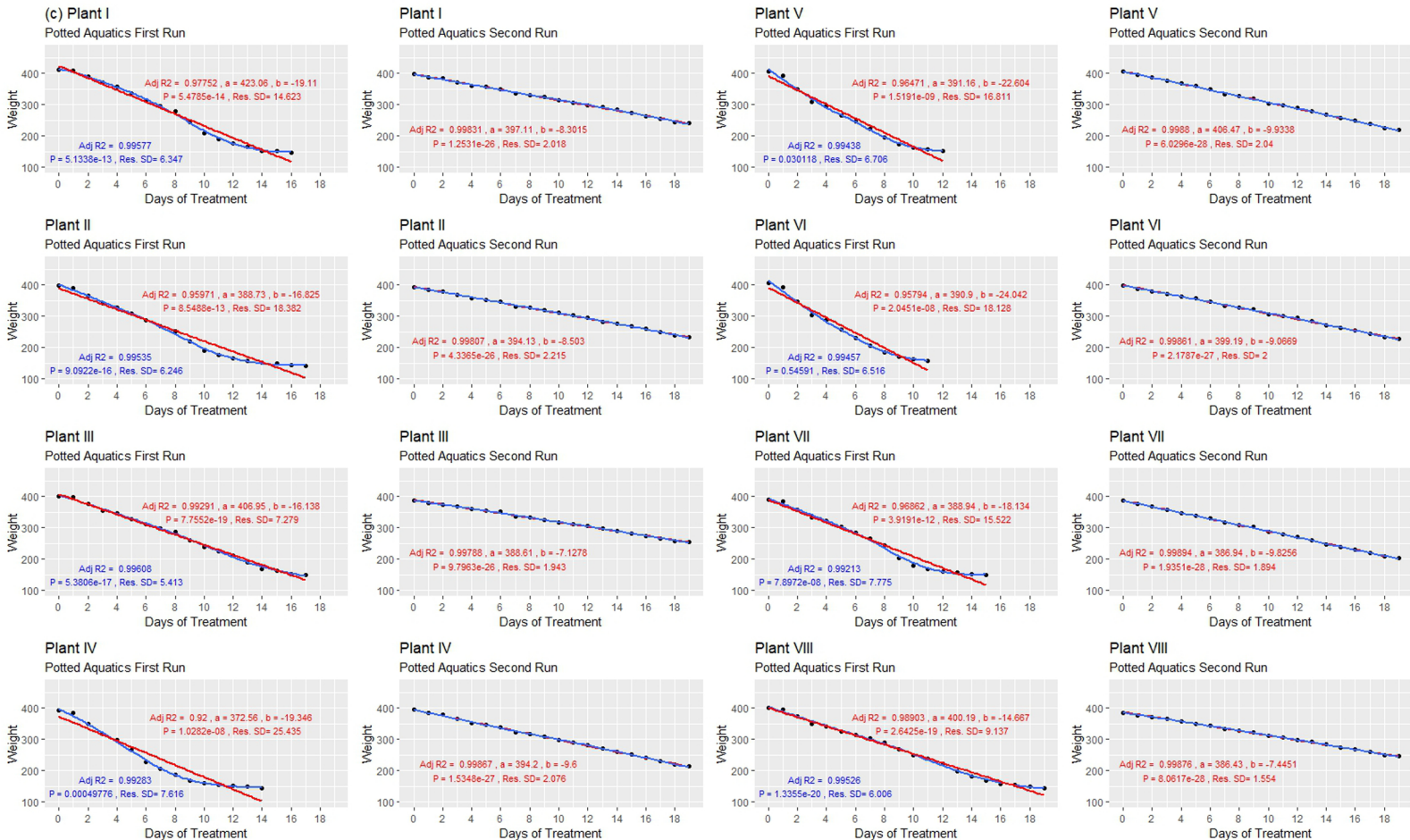

Supplement: Supplementary file 1 [file ECE3-9-13954-s001.zip › ece35833-sup-0003-FigS1c.pdf]
