# Supplementary material for: Ecotypes or phenotypic plasticity—The aquatic and terrestrial forms of Helosciadium repens (Apiaceae)
Source: Ecol Evol. 2019 Nov 25;9(24):13954–65. doi: 10.1002/ece3.5833 (PMC6953667; doi:10.1002/ece3.5833)
Supplement: Supplementary file 2 [file ECE3-9-13954-s002.docx]

Table S1: ISSR primer used in the analysis.

| **Primers** | **Motif 5´-3´** | **polym.** | **monom.** | **P%** | **PIC** |
| --- | --- | --- | --- | --- | --- |
| HB 11 | (GT)_6_CC | 7 | 4 | 63.6 | 0.3086 |
| HB 13 | (GAG)_3_GC | 9 | 7 | 56.3 | 0.3911 |
| HB 15 | (GTG)_3_GC | 4 | 5 | 44.4 | 0.2647 |
| UBC 807 | (AG)_8_T | 8 | 3 | 72.7 | 0.3721 |
| UBC 810 | (GA)_8_T | 8 | 5 | 61.5 | 0.4409 |
| UBC 811 | (GA)_8_C | 7 | 3 | 70.0 | 0.4207 |
| UBC 813 | (CT)_8_T | 14 | 4 | 77.8 | 0.3605 |
| UBC 834 | (AG)_8_YT | 7 | 11 | 38.9 | 0.3446 |
| total |  | 64 | 42 |  |  |
| avarage |  | 8 | 5.25 | 60.7 |  |

Polym.= polymorphous bands, monom.= monomorphous bands, P%= percentage of polymorphous band, PIC= Polymorphic information content.
