# Supplementary material for: Ecotypes or phenotypic plasticity—The aquatic and terrestrial forms of Helosciadium repens (Apiaceae)
Source: Ecol Evol. 2019 Nov 25;9(24):13954–65. doi: 10.1002/ece3.5833 (PMC6953667; doi:10.1002/ece3.5833)
Supplement: Supplementary file 3 [file ECE3-9-13954-s003.docx]

Table S2: Diversity parameters from the SSR analysis of *H. repens* in Germany (modified after Herden et al. (2019, unpublished data)).

| **Lab-ID** | **n** | **MLG** | **GT** | **All** | **rare** | **private** | **all.rich** | **F** | **Fis** | **mean delta** | **lat** | **cat** | **eco** |
| --- | --- | --- | --- | --- | --- | --- | --- | --- | --- | --- | --- | --- | --- |
| 1R | 27 | 11 | 20 | 16 | 0 | 0.037 | 1.870 | 0.336 | 0.462 | 0.3884 | 53.82 | N | Terr |
| 5R | 25 | 3 | 9 | 11 | 0 | 0 | 1.251 | 0.656 | 0.667 | 0.447 | 53.52 | N | Terr |
| 8R | 30 | 7 | 11 | 11 | 0.033 | 0 | 1.489 | 0.143 | 0.066 | 0.4104 | 51.72 | N | Terr |
| 9R | 30 | 6 | 12 | 12 | 0 | 0 | 1.568 | 0.330 | 0.206 | 0.2798 | 52.48 | N | Terr |
| 10R | 29 | 6 | 7 | 7 | 0 | 0.034 | 1.109 | -0.074 | -0.057 | 0.3798 | 54.31 | N | Terr |
| 12R | 26 | 3 | 8 | 8 | 0 | 0 | 1.140 | -0.040 | -0.031 | 0.3409 | 52.66 | N | Terr |
| 16R | 30 | 5 | 10 | 9 | 0 | 0 | 1.443 | 0.137 | -0.13 | 0.3155 | 47.54 | BY | Aqu |
| 18R | 27 | 22 | 18 | 17 | 0.037 | 0 | 2.051 | -0.012 | -0.016 | 0.2498 | 48.82 | BY | Terr |
| 19R | 30 | 5 | 9 | 10 | 0 | 0 | 1.631 | -0.241 | -0.464 | 0.3617 | 47.87 | BY | Aqu |
| 20R | 29 | 6 | 10 | 10 | 0 | 0 | 1.409 | 0.013 | 0.047 | 0.2966 | 47.94 | BY | Aqu |
| 21R | 30 | 10 | 14 | 13 | 0.067 | 0 | 1.920 | -0.184 | -0.177 | 0.4754 | 47.71 | BY | Aqu |
| 22R | 29 | 12 | 15 | 14 | 0 | 0.034 | 1.784 | 0.051 | 0.089 | 0.4902 | 47.67 | BY | Aqu |
| 24R | 29 | 8 | 11 | 12 | 0 | 0 | 1.515 | -0.329 | -0.617 | 0.3136 | 48.14 | BY | Aqu |
| 25R | 28 | 7 | 12 | 13 | 0 | 0 | 1.868 | -0.505 | -0.69 | 0.3845 | 48.13 | BY | Aqu |
| 27R | 26 | 20 | 30 | 18 | 0.038 | 0 | 2.599 | 0.350 | 0.245 | 0.268 | 47.83 | BY | Terr |

Lab-ID= working ID, n= sample size, MLG= multi-locus genotypes, GT= genotypes, All= alleles, rare= number of rare alleles per individual, private= number of rare alleles per individual, all.rich= average allelic richness, F= Fixation-Index, F_is_= F_is_-Index after Weir and Cockerham (1984), mean delta= compositional differentiation at genotype level, lat= decimal latitude values (WGS84), cat= category (BY= southern populations, N= northern populations), eco=ecosystem (Terr= terrestrial; Aqu= aquatic)
